# Multidimensional Rietveld refinement of high-pressure neutron diffraction data of PbNCN

**DOI:** 10.1107/S1600576724007635

**Published:** 2024-09-05

**Authors:** Yannick Meinerzhagen, Katharina Eickmeier, Peter C. Müller, Jan Hempelmann, Andreas Houben, Richard Dronskowski

**Affiliations:** a Institute of Inorganic Chemistry RWTH Aachen University 52056Aachen Germany; Tohoku University, Japan

**Keywords:** multidimensional Rietveld refinement, neutron diffraction, high-pressure studies, angular dispersion, wavelength dispersion, SNAP beamline, lead cyanamide

## Abstract

High-pressure neutron powder diffraction data from PbNCN were collected on the high-pressure diffraction beamline SNAP located at the Spallation Neutron Source (SNS) of Oak Ridge National Laboratory (Tennessee, USA). The diffraction data were analyzed using the novel method of multidimensional (two dimensions for now, potentially more in the future) Rietveld refinement and, for comparison, employing the conventional Rietveld method. To achieve two-dimensional analysis, a detailed description of the SNAP instrument characteristics was created, serving as an instrument parameter file, and then yielding both cell and spatial parameters as refined under pressure for the first time for solid-state cyanamides/carbodi­imides. The bulk modulus *B*_0_ = 25.1 (15) GPa and its derivative *B*′_0_ = 11.1 (8) were extracted for PbNCN following the Vinet equation of state. Surprisingly, an internal transition was observed beyond 2.0 (2) GPa, resulting from switching the bond multiplicities (and bending direction) of the NCN^2−^ complex anion. The results were corroborated using electronic structure calculation from first principles, highlighting both local structural and chemical bonding details.

## Abstract 2

The results are presented of a high-pressure neutron powder diffraction study on lead cyanamide, carried out with the novel method of multidimensional Rietveld refinement. In addition to better resolution than the standard one-dimensional case, the results show the first evidence of changing C—N bond multiplicities as a function of pressure, numerically quantified from density functional theory calculations.

## 1. Introduction

Multidimensional (two dimensions and beyond) Rietveld refinement is a novel method being developed for analyzing neutron powder diffraction data. Originally, the method targeted the time-of-flight diffractometer POWTEX (Conrad *et al.*, 2008 ▸; Houben *et al.*, 2012 ▸) presently being constructed at FRM-II in Garching near Munich (Germany). After preliminary analytic work (Jacobs *et al.*, 2015 ▸, 2017 ▸) and a successful test of the POWTEX detector (Modzel *et al.*, 2014 ▸; Houben *et al.*, 2023 ▸) on the POWGEN (Huq *et al.*, 2019 ▸) beamline at the Spallation Neutron Source (SNS) of Oak Ridge National Laboratory (Tennessee, USA) (Mason *et al.*, 2006 ▸) including some ‘shocking’ results, we are now extending the method to further instruments, possibly *all* time-of-flight neutron diffractometers worldwide, simply due to the fundamental nature of this approach. In the present case discussed here, we evaluate measured data from the SNAP beamline (Calder *et al.*, 2018 ▸; Frost *et al.*, 2020 ▸) at the SNS and highlight the multidimensional Rietveld performance using a typical solid-state chemistry example, PbNCN, lead cyanamide.

As solid-state cyanamides and carbodi­imides define a relatively new class of compounds, their high-pressure behavior has not been investigated extensively; the literature only covers studies of HgNCN, PbNCN and BaNCN (Masubuchi *et al.*, 2022 ▸; Liu *et al.*, 2002 ▸; Möller *et al.*, 2018 ▸). For PbNCN, Möller *et al.* (2018 ▸) started with powder XRD studies and further predicted a few unexpected structural changes at high pressures on the basis of density functional theory (DFT) calculations. This prompted us to choose PbNCN as our object of study and to investigate its behavior under pressure, but with a better structure-analytic probe, namely time-of-flight neutron diffraction.

**Figure 1:**
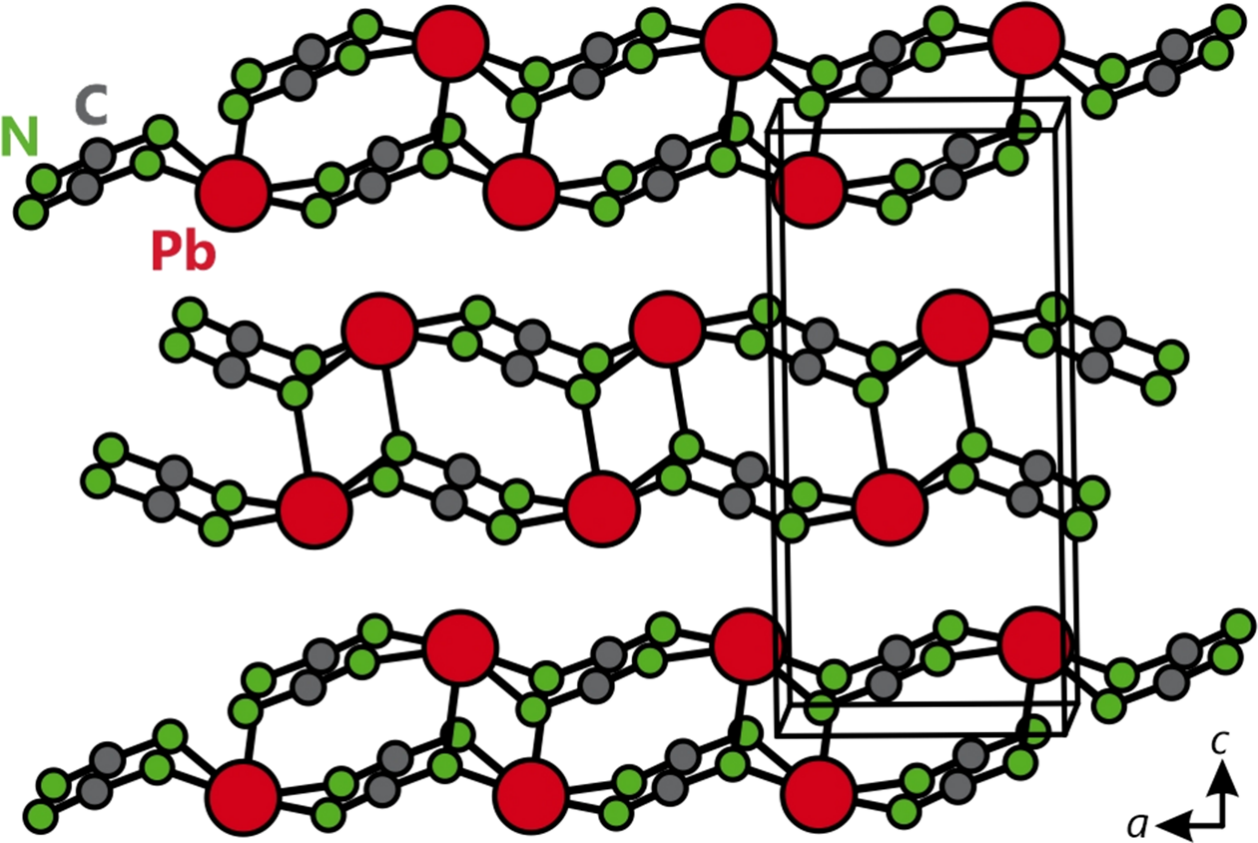
The layered structure of PbNCN with lead atoms in red, carbon in dark gray and nitro­gen in green.

Under standard conditions, lead cyanamide crystallizes in the orthorhombic space group *Pnma* with unit-cell parameters of *a* = 5.5566 (4), *b* = 3.8677 (2) and *c* = 11.7350 (8) Å (Liu *et al.*, 2000 ▸) and a cell volume of 252.20 (15) Å^3^. The crystal structure consists of Pb—NCN chains along the *a* axis and corrugated double ‘layers’ of NCN^2−^ and Pb^2+^ in the *ab* plane (Fig. 1 ▸). The divalent lead cation is coordinated in a distorted square-pyramidal shape [1 × 2.31 (2) Å, 4 × 2.62 (2) Å] with two additional, presumably nonbonding, nitro­gen neighbors augmenting the coordination sphere at 3.43 (2) Å, so the coordination number is approximately 5 + 2 = 7.

Within PbNCN the linear NCN^2−^ complex anion exhibits, due to being bonded to the Pearson soft Pb^2+^ cation, two significantly different bond lengths [1.30 (3) and 1.16 (3) Å] and is therefore called a *cyanamide* anion with an N—C single and a C≡N triple bond, N—C≡N^2−^. There are also plenty of solid-state *carbodi­imides* in which the complex anion adopts an N=C=N^2−^ shape, with two N=C double bonds at about 1.22 Å. In all of these phases, the NCN^2−^ complex anion somehow serves as a ‘divalent nitride’ (N^2−^ instead of the correct N^3−^). Therefore, NCN^2−^ may replace the O^2−^ oxide anion chargewise, but with a more covalent bonding character to whatever metal cation, simply because nitro­gen is less electro­negative than oxygen.

## 2. Methods

### 2.1. Synthesis

Lead cyanamide was obtained from the reaction of lead acetate and an aqueous solution of molecular cyanamide, following the synthesis described by Liu *et al.* (2000 ▸). An ammonia solution was added to a mixture of lead acetate and cyanamide to obtain a pH of 10 and to start precipitation of PbNCN. It crystallizes as a yellow powder which was filtered off, washed with water and dried. To collect powder X-ray diffraction patterns, the sample was ground and fixed with grease on acetal sheets which were held between split aluminium rings. Data were collected at room temperature with Cu *K*α_1_ radiation (λ = 1.54059 Å) using a Stoe StadiP diffractometer (Stoe & Cie GmbH, Darmstadt, Germany) equipped with a MYTHEN 1K detector. The raw data were processed using the *WinXPow* (Stoe & Cie, 2008 ▸) software package, revealing that lead cyanamide had been obtained as a phase-pure material.

### 2.2. Measurement strategy

The *in situ* high-pressure measurements were conducted at Oak Ridge National Laboratory (Oak Ridge, Tennessee, USA) using the VX5 Paris–Edinburgh (PE) press on the SNAP high-pressure diffractometer. The powder was pressed into a spherical pellet and transferred into the PE press with a MeOH/EtOH (4:1) mixture as the hydro­static medium. The pressure was indirectly controlled via an external oil pressure operating on two anvils. Because the true sample pressure was not directly accessible by the instrument setup, a second sample containing a mixture of PbNCN (72.7%, 218.5 mg) and lead (27.3%, 82.3 mg) was measured and thereby served as a calibrant in the same range of oil pressures. The high-pressure behavior of lead has been described in full detail by Strässle *et al.* (2014 ▸), with a bulk modulus of *B*_0_  = 41.20 (20) GPa and a pressure derivative of 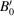 = 5.72 (20) at 300 K. Hence, the pressures inside the sample could be straightforwardly determined according to the Vinet equation of state (Vinet *et al.*, 1987 ▸), given as
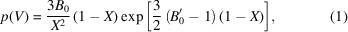
with the definition of 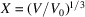, the bulk modulus at ambient pressure given as 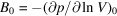 and its pressure derivative at ambient pressure being 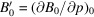.

### 2.3. DFT calculations

All DFT-based calculations were executed using the *Vienna Ab Initio Simulation Package* (*VASP*) (Kresse & Furthmüller, 1996*a* ▸,*b* ▸; Kresse & Hafner, 1993 ▸) with projector-augmented waves (Blöchl, 1994 ▸) for the pseudopotential setup and the GGA-like PBEsol functional (Csonka *et al.*, 2009 ▸) for exchange and correlation. Additionally, a D3 correction with Becke–Johnson damping (Grimme *et al.*, 2010 ▸, 2011 ▸) was employed to account for weaker dispersion interactions, likely to be present here taking into account the ‘layered’ motif of PbNCN. The reciprocal **k**-point meshes were created in accordance with the Monkhorst–Pack scheme (Monkhorst & Pack, 1976 ▸) and considered **k**-converged at 11 × 15 × 5. The integration of the Brillouin zone was done using Blöchl’s tetrahedron method (Blöchl *et al.*, 1994 ▸). The essentially converged plane-wave energy cutoff was defined at 500 eV. The present approach resembles what has been done before (Möller *et al.*, 2018 ▸), although the exchange–correlation functional and dispersion correction used now may be slightly more accurate.

Because of a persistent DFT problem (see below), structural optimization was only applied to the unit-cell volume for achieving a reasonable ground state, and the convergence criterion was 10^−8^ eV for electronic steps and a residual force of 10^−3^ eV Å^−1^ for the ionic steps. Since PbNCN involves different C—N bond multiplicities, even gradient-corrected functionals such as PBEsol (which all suffer from the DFT-typical delocalization error) cannot reliably serve to model correctly the shape of such anions (N≡C—N^2−^ versus N=C=N^2−^ versus N—C≡N^2−^) (Liu *et al.*, 2003 ▸), so the final DFT-based chemical-bonding analyses were therefore based on the experimental internal positional parameters from neutron diffraction, *i.e.* without further optimization. To do so, *LOBSTER* (Maintz *et al.*, 2013 ▸, 2016 ▸; Müller *et al.*, 2021 ▸; Nelson *et al.*, 2020 ▸) was used to project the plane-wave-based wavefunctions resulting from DFT onto a local orbital basis and calculate the crystal orbital bond index (COBI) (Müller *et al.*, 2021 ▸).

### 2.4. Data reduction

Both the multidimensional and the conventional data treatment were carried out with the same raw event data. In order to perform a multidimensional (two dimensions in this case) Rietveld refinement, the diffraction data were first reduced using the *Mantid* software (Arnold *et al.*, 2014 ▸), following a similar approach to that described by Houben *et al.* (2023 ▸). Therefore, the publicly available *Mantid* algorithm *PowderReduceP2d* was adapted following the SNAP instrument-specific calibration method. This algorithm yields coordinates in the strictly orthogonal (*d*, *d*_⊥_) diffraction space, just like in the known .p2d file format being read by our modified version of the *GSAS-II* software (Toby & Von Dreele, 2013 ▸). The modifications to *PowderReduceP2d* were released with *Mantid* Version 6.8 (Mantid Project, 2023 ▸). For technical reasons, we only used the measuring data collected with one of the two detectors; further details are specified in the supporting information.

### 2.5. Data refinement

The 2D refinements were accomplished with a modified in-house version of *GSAS-II* as previously used by Houben *et al.* (2023 ▸). The modified algorithms are based on the developments introduced by Jacobs *et al.* (2015 ▸, 2017 ▸). The conventional (1D) refinements were done with SVN revision 5136 of *GSAS-II* (Toby & Dreele, 2013 ▸). For all single-phase refinements, the following scheme was applied for the order of refined parameters: (i) scale factor, (ii) unit-cell parameters, (iii) spatial parameters, and (iv) unit-cell and spatial parameters together. For the two-phase refinements, the order was (i) individual scale factors, (ii) unit-cell parameters (phase 1), (iii) unit-cell parameters (phase 2), (iv) scale factors together and (v) unit-cell parameters together. In the case of the 2D approach the instrument parameter DthF as given in Table 1 was refined once for the PbNCN single-phase measurement at the lowest pressure. The resulting value was used for all following refinements as the instrument does not change during the measurements.

All observed and calculated data points used with the conventional and multidimensional refinements are provided as supporting information to this article using the CIF format. Below the structural model, the final section of each CIF contains several columns with the column heads given at the beginning and then looping over all data points. The first four columns of each line hold the position of the data point in (2θ, λ) and (*d*, *d*_⊥_) coordinates. Since the *d*_⊥_ coordinate is not (yet) known to the CIF format, the corresponding column is named by the keyword _pd_proc_ls_special_details. Further columns list the observed (_pd_meas_intensity_total), calculated (_pd_calc_intensity_total) and background (_pd_proc_intensity_bkg_calc) intensities. The .cif files for the conventional data sets follow a similar format.

## 3. Results and discussion

### 3.1. The SNAP instrument and multidimensional data treatment

The design philosophy of SNAP corresponds to a high-flux medium-resolution neutron diffractometer dedicated to high-pressure experiments, so it allows easy sample access given all sorts of pressure cells with adjustable shielding to minimize background (Calder *et al.*, 2018 ▸). Although the angular coverage of the two Anger camera detectors of SNAP is smaller than that with general-purpose powder diffractometers with large-area detector coverage such as POWGEN (SNS) or future instruments like POWTEX (MLZ, Germany) or DREAM (ESS, Sweden) (Schweika *et al.*, 2016 ▸), on purpose we apply the same strategy of a fundamental resolution description to SNAP as introduced by Jacobs *et al.* (2017 ▸). Since the SNAP instrument configuration is very flexible and allows different pressure cells to be accommodated, *i.e.* different sample volumes, different aperture settings *etc.*, the derived assumptions fit mainly to our experimental setup. The detailed explanation in the next section especially addresses (SNAP) users planning to use a multidimensional data treatment with principally similar but slightly different setups, since that is mandatory for a multidimensional *GSAS-II* Rietveld refinement.

We will first show that there is an advantage in applying this method more generally to other time-of-flight (TOF) diffractometers (such as SNAP) in addition to those for which it was developed. Second, we also point out that considering the instrument description as part of the experiment is highly important, as the description can strongly influence the scientific evaluation of the measured data.

### 3.2. Multidimensional instrumental parameter description for SNAP

The fundamental parameters for calculating the instrumental resolution function for SNAP will be derived using the same formalism as used for POWGEN by Jacobs *et al.* (2017 ▸), but on the basis of the current design parameters of SNAP and according to the experiment performed. These parameters were taken either from Calder *et al.* (2018 ▸), from the SNAP homepage at the SNS, or from privately communicated information by the instrument scientists Antonio Moreira dos Santos and Malcolm Guthrie.

The primary flight path from source to sample is set to *L*_1_ = 15 m within the ≲10 cm wide neutron guide, while the sample-to-detector distance is constantly *L*_2*A*_ = 0.5 m for all measurement positions of the detector center (the flat detector is a tangent, with its center at the contact point with the 2θ circle and with its surface being normal to the scattered neutron beam lying in the horizontal scattering plane). Taking this geometry and the detector dimensions (see below) into account, one can calculate the average sample-to-detector distance to be *L*_2Avg_ ≃ 0.6 m. The parameter *L*_2*B*_ – used to describe the equi-angular spiral-like detector layout of POWGEN (Huq *et al.*, 2019 ▸) – is not applicable and is set to *L*_2*B*_ = 0 rad^−1^. Thus, the secondary flight path arrives at a constant *L*_2_ = *L*_2*A*_ for the detector center placed at 2θ = 65°. We used a Paris–Edinburgh cell, with a spherical sample of ∼5 mm diameter being the main effective contribution to the uncertainty in length. A Gaussian sample width of *w*_sample_ = Δ*L* = 3.6 mm (FWHM) is yielded for a sphere being projected onto the detector surface. For ambient pressures, this gives a relative length uncertainty of
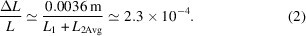


The time uncertainty is mainly determined by the SNS moderator and, to a first approximation, is proportional to the neutron wavelength and thus also to the total time of flight. With an estimate of Δ*t* = 10 µs and an averaged total time of flight *t* = 3944 µs for a 2θ-averaged flight path of *L* ≃ 15.6 m and for a wavelength of λ = 1.0 Å, then independent of wavelength one obtains the constant relative time resolution
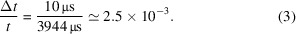


As described by Frost *et al.* (2020 ▸), the horizontal beam divergence simulated for the chosen ‘middle divergence’ setup was Δ2θ_div_ = 17.69 mrad. The angular resolution of the Anger camera detector can be calculated from the work of Calder *et al.* (2018 ▸). The 3 × 3 detector array has an edge length of 0.45 m. With 256 channels per detector per edge this gives as a quotient a pixel width of 0.59 mm, with a continuous uniform distribution equivalent to a Gaussian FWHM of *w*_detector_ ≃ 0.41 mm. As above, the Gaussian sample width at ambient pressure results in *w*_sample_ = 3.6 mm (FWHM). From these, the angular uncertainties are calculated by
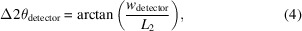

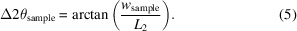


By applying equation (16) of Jacobs *et al.* (2017 ▸), we arrive at a relative resolution of Δ*d*/*d* = 8.55 × 10^−3^ at 2θ = 90°, which is slightly larger than the value of Δ*d*/*d* = 8 × 10^−3^ given by Calder *et al.* (2018 ▸) for 2θ = 90° without specifying the sample size. As already alluded to above, this instrument parametrization *specific to our setup* serves us well enough, in particular as we mainly focus on the unit-cell and spatial parameters as a function of pressure. Instrumental parameters like difC [the linear conversion factor relating the measured TOF and the corresponding *d* value; see Houben* et al.* (2023 ▸)] and the raise/decay terms of the back-to-back exponential part of the profile function, α and β, were taken from the conventional instrument parametrization. Only non-fundamental resolution parameters, such as *u*_1_ (now dubbed DthF) and Δ_add_ as used by Jacobs *et al.* (2017 ▸), are refineable during Rietveld analysis and also reveal the quality of the fundamental instrumental description when deviating from their expected values (DthF = 1.0; Δ_add_ = 0.0).

**Figure 2:**
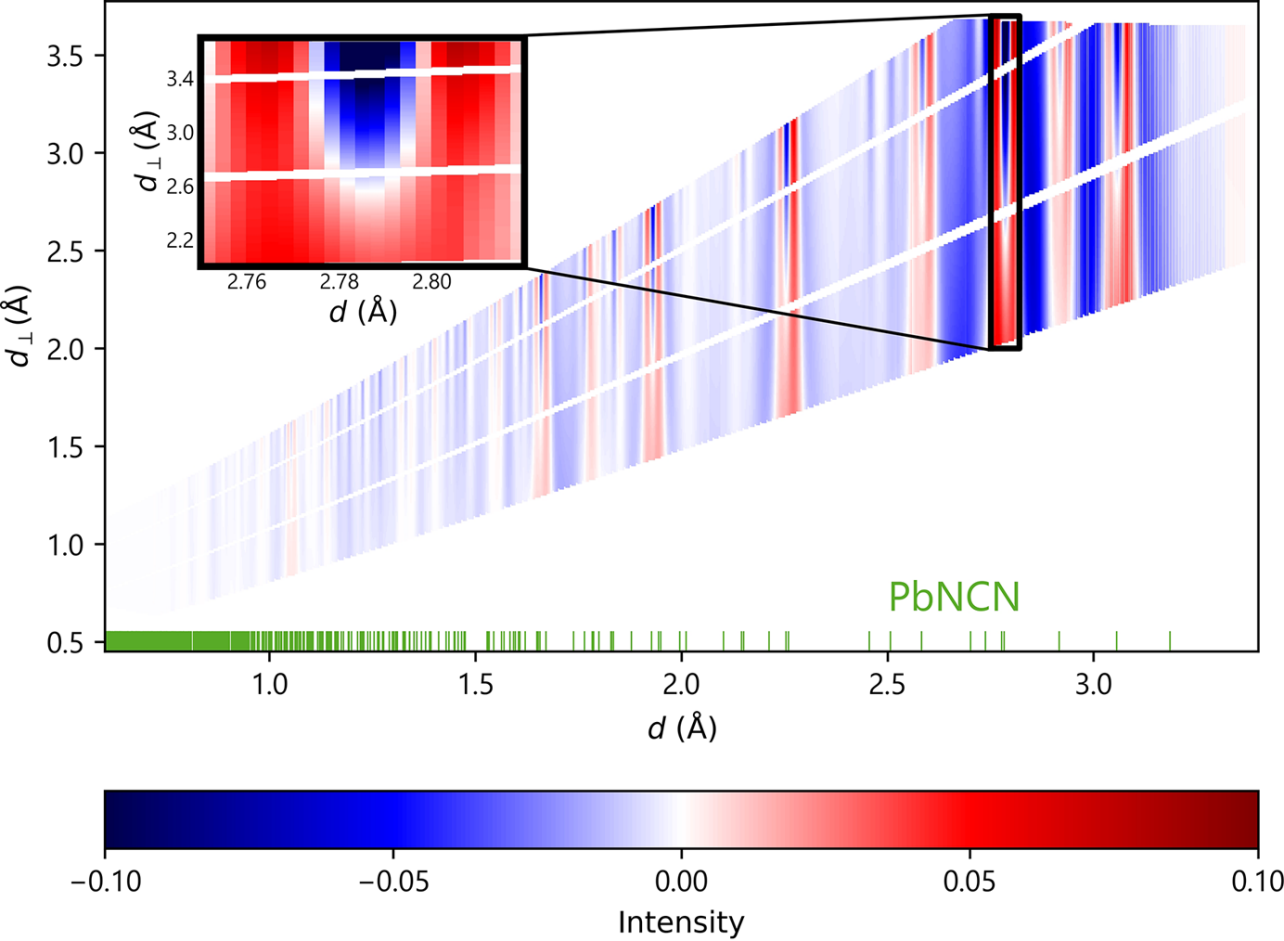
A difference plot of the difference plots of the 2D refinements done with preliminary and final instrument parameters. The highlighted area is one of many that shows a substantial change in the intensities depending on *d*_⊥_.

To emphasize the importance of a well described instrument, we compare the refinement results of the first pressure point (single-phase measurements) using the final instrumental description with the same data refined with preliminary instrumental parameters which were based on an initial and still limited instrumental description. To visualize the improvement made, the difference plot obtained with the preliminary parameters was subtracted from the difference plot of the final parameters (Fig. 2 ▸). It is immediately obvious that a slight change in instrument parameters makes a significant difference for the refinement model. The highlighted area of Fig. 2 ▸ shows as an example the much better description using the new instrument parameters concerning the peak width at high *d*_⊥_ values (which correspond to high 2θ and λ of a constant *d* reflection, *i.e.* the backscattering regime). This behavior can be traced back mainly to the amendment of the time resolution Δ*t*/*t* (from 0.0028 to 0.0025). Further changes to the parameters contributing to the angular un­certainty result in an improved value for the correction term DthF from 0.67 (1) to 0.854 (4). Again, the target value for DthF is 1.0 according to Jacobs *et al.* (2017 ▸), meaning that, ideally, all effects contributing to the multidimensional resolution function are perfectly described and the derived parameters are not refined in the Rietveld analysis. If the deviations from these expected values are too strong, the instrumental description should be reconsidered instead. In this case, the above parameters predominantly influence the peak width for the low *d*_⊥_ range (the influence increases in the forward scattering regime, *i.e.* low 2θ, where the time resolution becomes less important) and the resulting peak width is already sufficiently accurate (Fig. 2 ▸). The optimized instrument parameter file led to a slightly improved *R*_Bragg_ value of 8.67%, compared with 8.74% previously. For some cell and spatial parameters, the accuracy improves by up to 0.5%. A comparison of all relevant preliminary and final instrument parameters is provided in Table 1 ▸.

The aforementioned results not only underline the importance of a well described instrument; they further illustrate the advantage of a 2D refinement using a fundamental instrument description. That is to say, certain refinement ambiguities indicated before only became visible and could be assigned to their origin in a two-dimensional plot. We expect the same approach also to enable an improved refinement of structural, physical and chemical models.

### 3.3. Sample pressure determination

Once all the instrument parameters were identified, the subsequent Rietveld refinements were performed on this basis.

**Figure 3:**
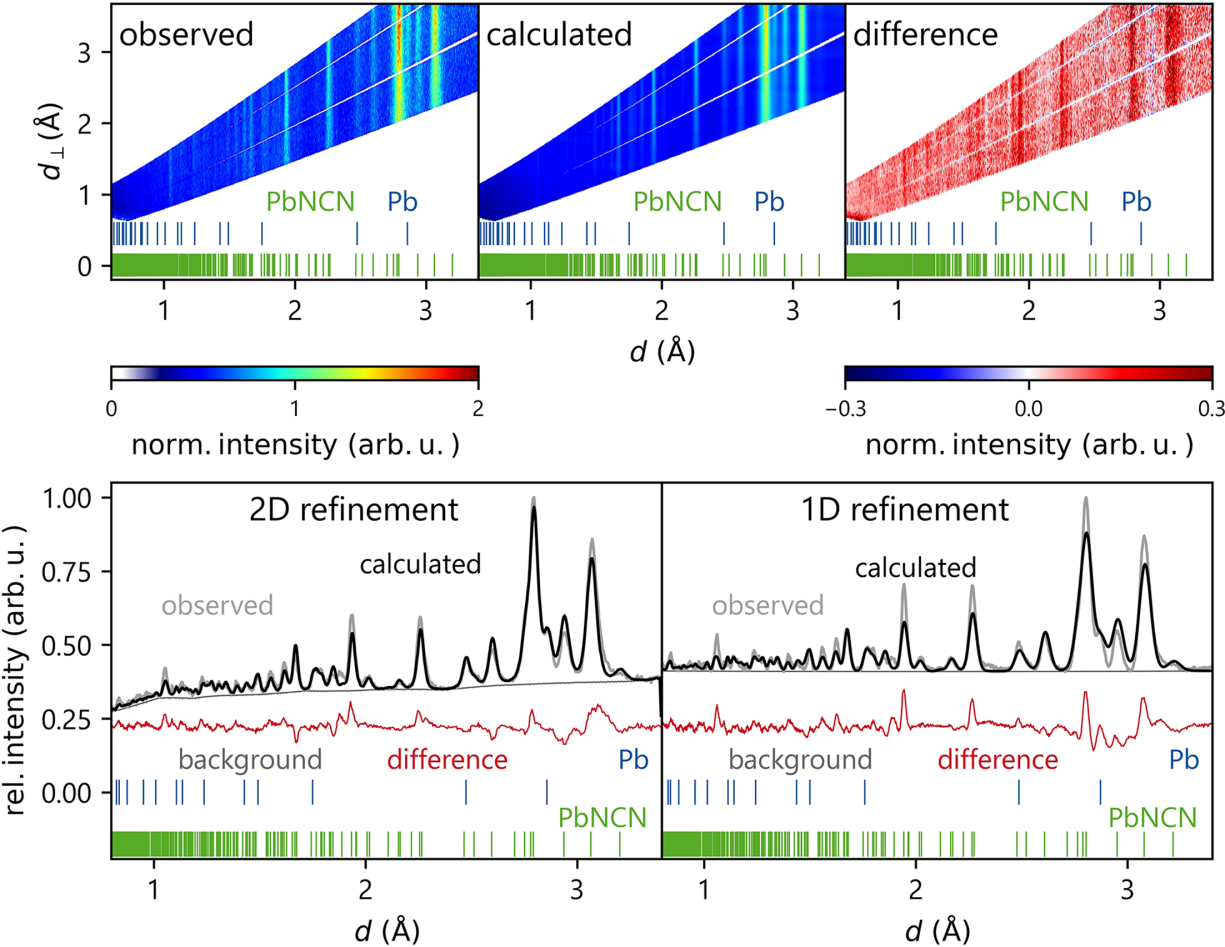
The top row from the left shows the observed, calculated and difference data of the 2D refinement for the lowest-pressure two-phase measurement. The bottom left plot shows the 2D refinement reduced to a projected 1D plot as a comparison with the results of the conventional 1D refinement (bottom right). Blue vertical lines indicate Pb reflection positions and green vertical lines indicate PbNCN reflection positions.

Fig. 3 ▸ depicts the results of the 2D refinement for the first pressure point of the two-phase sample, including both PbNCN and Pb. Incidentally, this is the first time a *two-phase* refinement has been presented with the novel method of multidimensional Rietveld refinement. The top row shows two-dimensional data representations of the observed and calculated data and the difference plot. The bottom left shows the 2D data displayed as a projected 1D plot for an easier comparison with the conventional 1D refinement plotted in the bottom right. The one-dimensional representation of the 2D refinement immediately manifests the superior description of the overlapping reflections around 3 Å compared with the 1D refinement. This relation between 1D and 2D refinements will also become apparent in the following parts of this work.

The sample pressures were each calculated from lead’s unit-cell volume change as refined from the corresponding two-phase Rietveld refinements following equation (1). The results for all volumes and the corresponding pressures can be found in Table S1 of the supporting information. The volumes of PbNCN and the sample pressures obtained from the two-phase refinements were then utilized to calculate values for the bulk modulus *B*_0_ and its pressure derivative 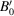 according to the model of Vinet *et al.* (1987 ▸). The results are *B*_0_ = 25.1 (15) GPa and 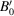 = 11.1 (8) for the 2D refinements and *B*_0_ = 18.9 (13) GPa and 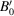 = 15.5 (9) for the 1D refinements. The 2D results in particular are quite similar to the bulk modulus *B*_0_ = 23.1 (3) GPa and its derivative 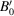 = 7.0 (3) of the structurally related and isoelectronic phase α-PbO (Giefers & Porsch, 2007 ▸). We note that the crystal structure of PbNCN can be derived from the PbO structure by a *translationen­gleiche* transition of index 2 and a *klassengleiche* transition of index 2.

Overall, the 2D refinement arrives at a volume change for lead from 121.2 (1) to 102.8 (4) Å^3^, and the corresponding PbNCN volumes reach from 252.59 (4) to 212.81 (16) Å^3^. The 1D refinement yields volume changes for lead from 123.06 (24) to 103.32 (10) Å^3^ and for PbNCN from 255.7 (10) to 215.6 (4) Å^3^. Thus, PbNCN’s less reliable 1D refinement result is larger than the 2D result by around 3 Å^3^ (see also below). In molar units, this corresponds to a difference of about 0.5 cm^3^ mol^−1^.

As the lead volume of the 1D refinement for the lowest pressure point [123.06 (24) Å^3^] is significantly larger than the literature value at ambient pressure [121.29 (8) Å^3^], we decided to use the pressure values calculated from the 2D refinements for further interpretation and all plots. The lead volumes for the lowest non-ambient pressure of the 2D refinements [121.2 (1) Å^3^] are slightly lower than the literature value at ambient pressure, as expected.

After having calculated the bulk modulus and its derivative for PbNCN using the two-phase sample, the pressure inside the single-phase sample became accessible as a function of the oil pressures, again leveraging the Vinet equation of state (Vinet *et al.*, 1987 ▸). The results are provided in Table S2 of the supporting information. As found for the two-phase sample, the PbNCN volumes from 1D refinements are roughly 3 Å^3^ (or 0.5 cm^3^ mol^−1^) larger than their 2D counterparts.

### 3.4. Single-phase refinement of PbNCN

**Figure 4:**
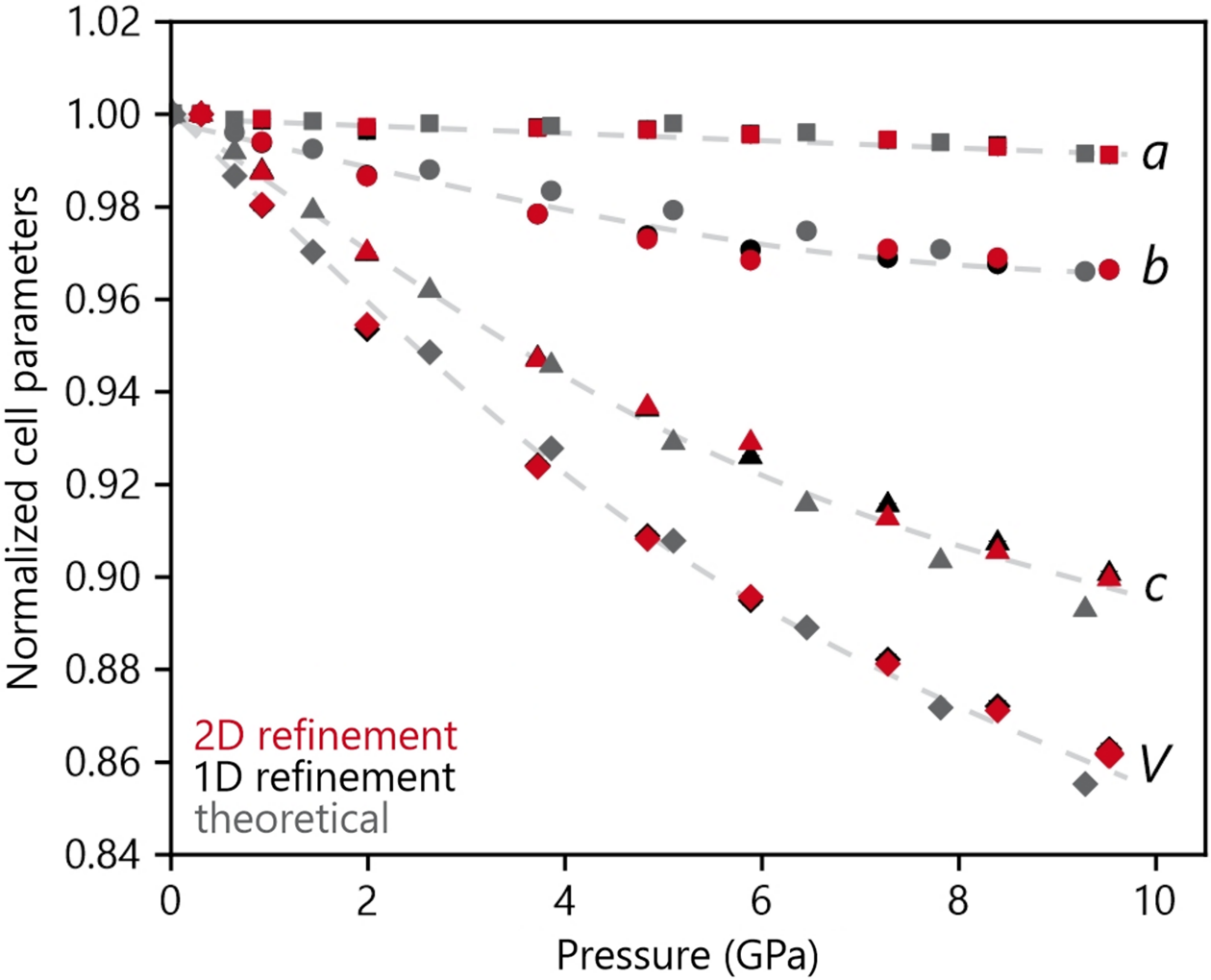
Normalized cell parameters for the 1D (black) and 2D (red) refinements, compared with theoretical data (gray). Error bars if not visible are inside the markers.

Fig. 4 ▸ shows the progression of the cell parameters and the volume, each normalized by the corresponding value of the first pressure point at 0.31 (2) GPa. The standard deviations are so small that they fall inside the markers already, and they are generally even smaller for the 2D refinements by around one order of magnitude, which is remarkable. The overall trends of the 1D and 2D refinements are the same. The numerical values of the cell parameters can be found in Table S4 of the supporting information.

**Figure 5:**
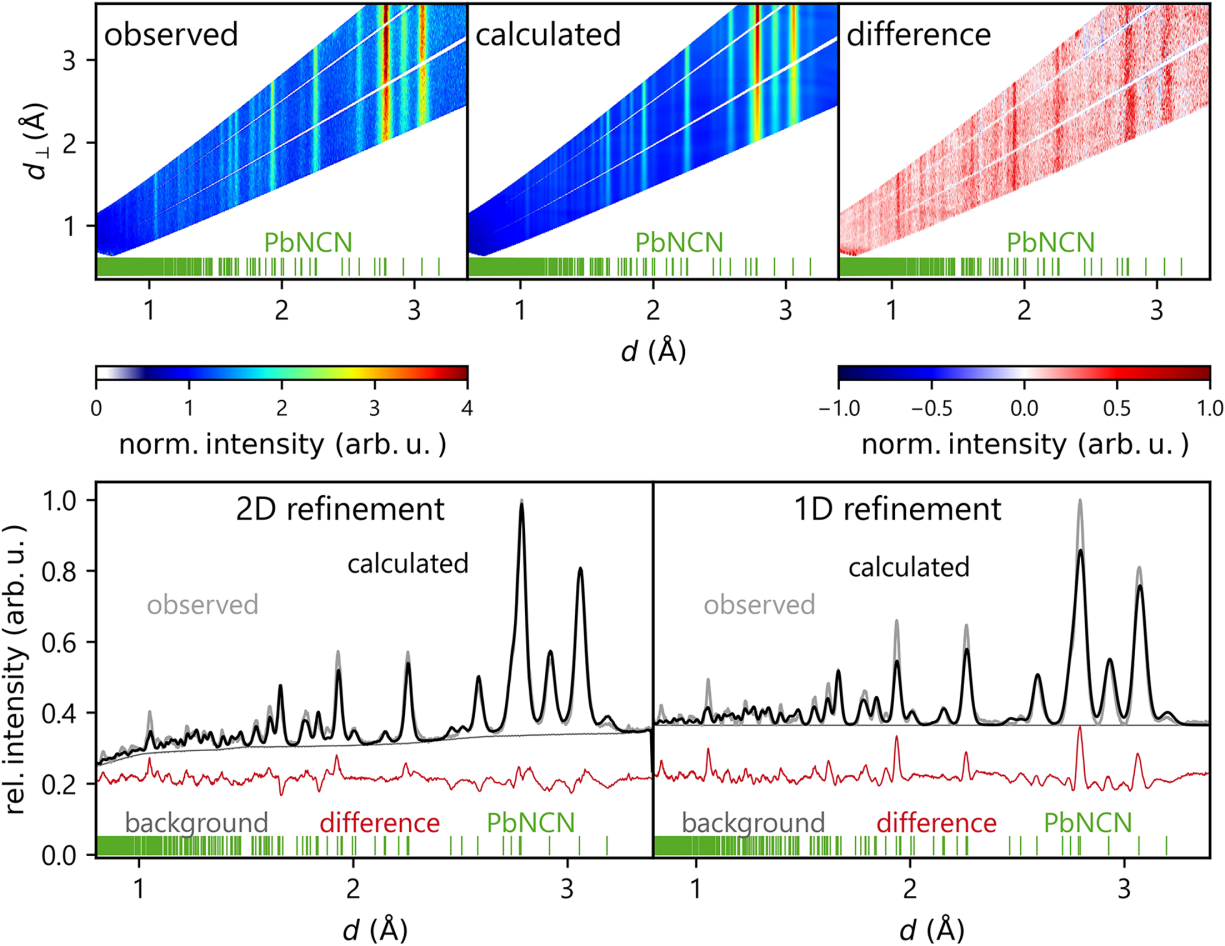
As in Fig. 3 but for the single-phase measurement. Green vertical lines indicate PbNCN reflection positions.

Fig. 5 ▸ shows as an example the refinement result for the lowest-pressure single-phase refinement, in the same style as given before (Fig. 3 ▸). One particular advantage of the 2D refinement becomes apparent when considering the strongest reflection at 2.78 Å: here, the 1D plot of the 2D refinement matches the observed data significantly better than the 1D-refined pattern. A detailed comparison of the spatial positions for all pressure points is given in Table S3 in the supporting information. As alluded to above and generally shown in the following, the standard deviations for the 1D refinements are always significantly larger than the 2D standard deviations, sometimes by an entire order of magnitude, for both unit-cell and spatial parameters. As they are calculated with the covariance matrix of the least-squares algorithm and the GOF (goodness of fit), which is directly affected by the degree of freedom, the substantially larger number of points in the 2D data set should not influence the difference in standard deviations this much. The GOF in *GSAS-II* is calculated as
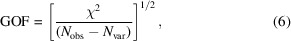
with χ^2^ the sum of the difference between the observed and calculated intensities for each data point, *N*_obs_ the number of observed points and *N*_var_ the number of refined parameters.

**Figure 6:**
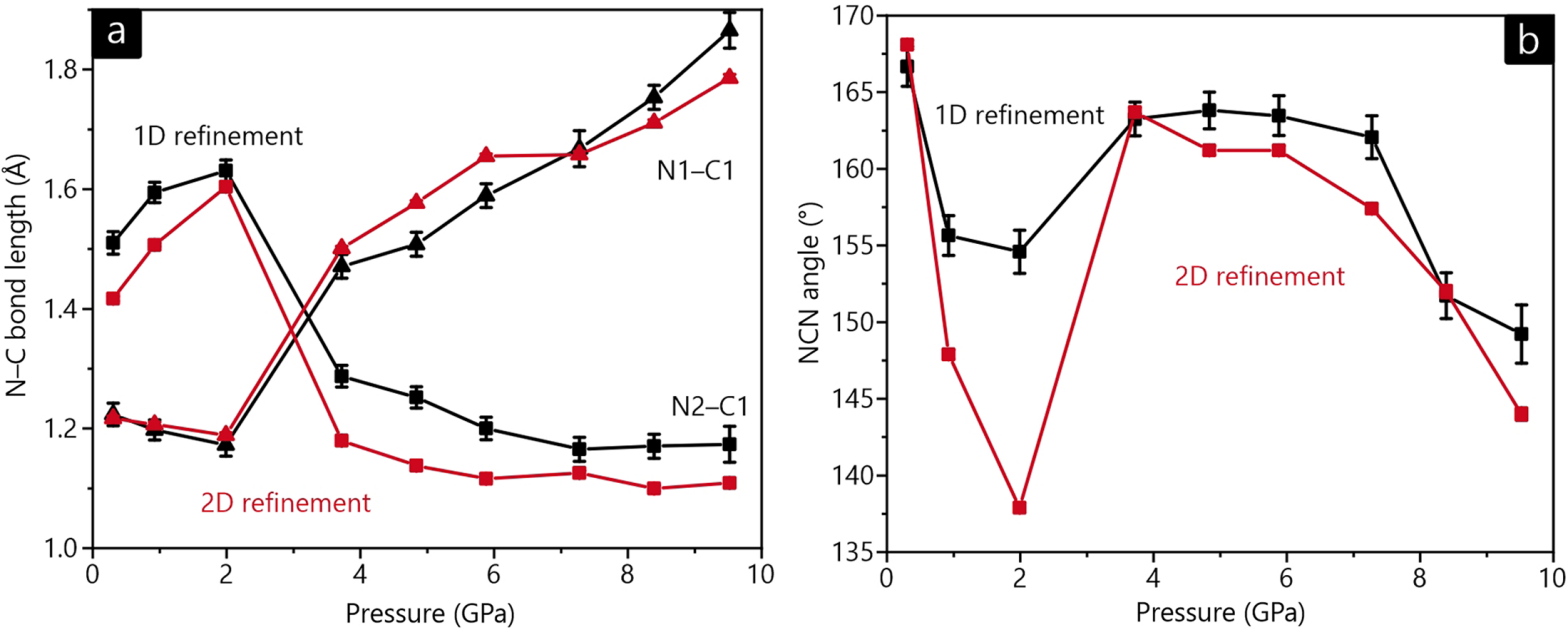
(*a*) The N—C bond lengths of the cyanamide unit for the 1D (black) and 2D (red) refinements. (*b*) The N—C—N angle as a function of pressure. If not visible, error bars are smaller than the markers.

**Figure 7:**
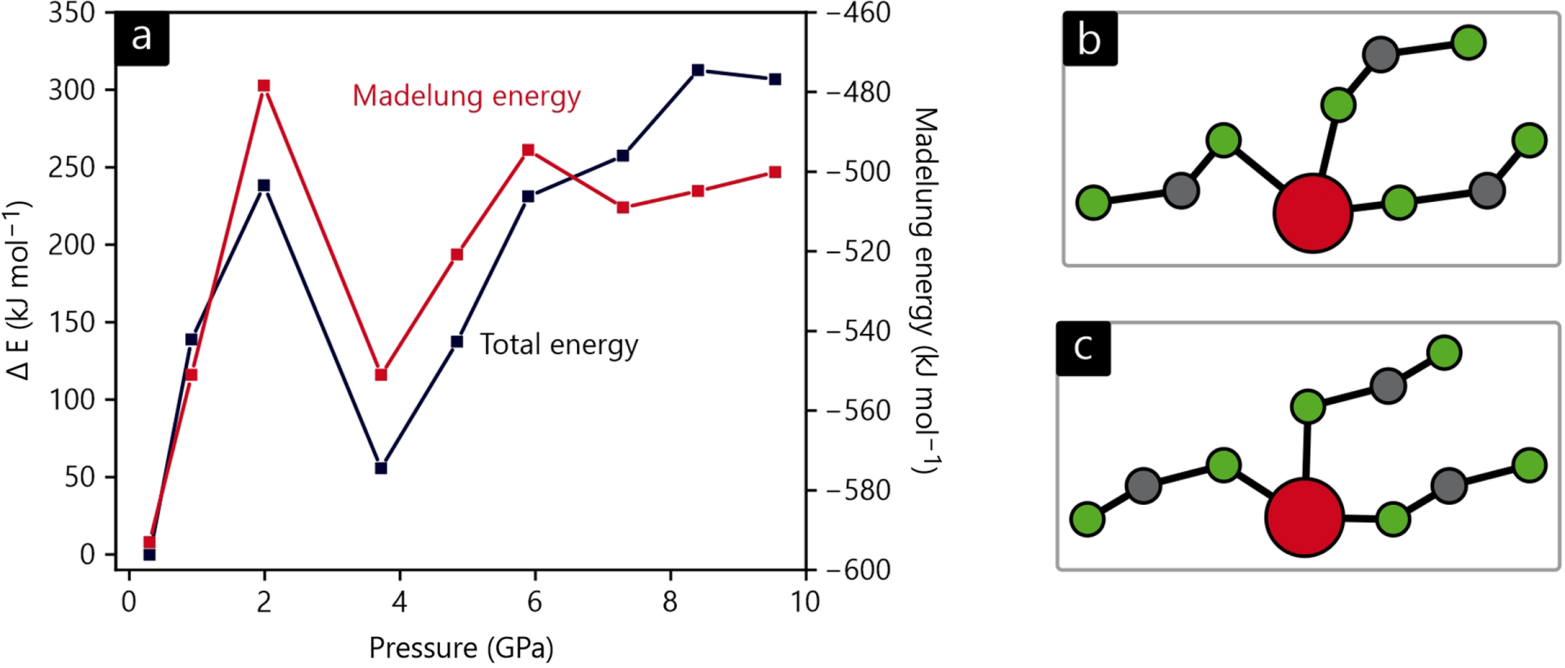
(*a*) The DFT total energy and Madelung energy versus pressure for the structures found by the 2D refinement. The DFT energy is given relative to the first pressure point. (*b*) The local Pb coordination at 2.0 (2) GPa and (*c*) at 3.7 (4) GPa.

We now take a closer look at the bond lengths and angle of the NCN^2−^ unit, as well as the environment of the lead atom. Fig. 6 ▸ displays the changes in the N—C bonds and the N—C—N angle with increasing pressure. It is immediately obvious, first, that something unexpected happens after the third pressure point as regards bond distances and, second, that the 1D and 2D refinements differ substantially at the second and third pressure points as regards the angle. In more detail, the short (triple) N≡C and long (single) C—N bonds *switch positions*, which is seemingly coupled with a soft ‘reset’ of the N—C—N angle to a value of 163° (the initial value was 168°). The general trend before and after this reset is comparable for both 1D and 2D refinements but is more pronounced in two dimensions: the N—C—N angle becomes more acute and the difference in the N—C bond lengths increases. This reset of the N—C—N angle is coupled with an additional internal change, namely a reorientation of the NCN^2−^ complex anion such that the central carbon atom is now pointing towards the layers themselves [Fig. 7 ▸(*c*)], whereas it was tilted towards the gaps between layers before [Fig. 7 ▸(*b*)].

To reduce the likelihood that the observed differences between the 1D and 2D refinements are due to being trapped in a local minimum, we used the 1D-refined structure as an initial guess for the 2D refinement and *vice versa*. Doing that, however, did not change the final results at all – the structural behavior is real.

In order to investigate these structural peculiarities more closely in terms of electronic structure theory, we performed total-energy calculations without structural optimization for each pressure point based on the 2D-refined structures. The resulting DFT energy differences and electrostatic Madelung energies are shown in Fig. 7 ▸(*a*). As expected, the energy rises during compression, at least for the first three data points. When going from 2.0 (2) to 3.7 (4) GPa (after the third pressure point), however, a change in the NCN^2−^ anionic shape takes place, pictured in Figs. 7 ▸(*b*) and 7 ▸(*c*), and this lowers the total energy by almost 200 kJ mol^−1^. After this jump, the total energy again rises constantly. This overall trend is also mirrored by the Madelung energy (except for the last three pressure points), so this simplified point-charge model almost gets it right, despite covalency becoming more important for the highest pressure.

**Figure 8:**
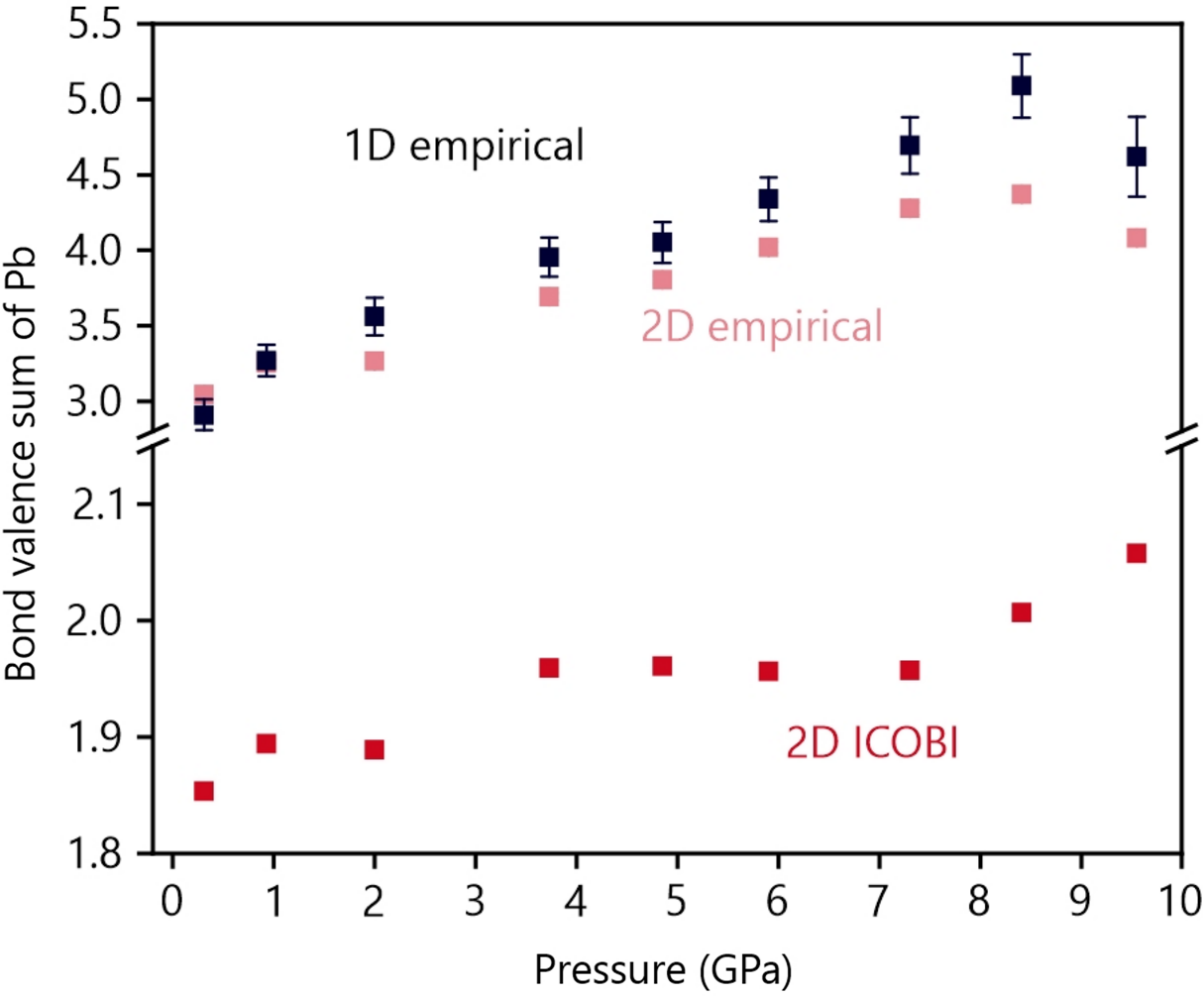
Bond-valence sum (dubbed empirical) of lead for the 1D (black) and 2D (light red) refinements, and the sum over the respective ICOBI (red) based on the structures from the 2D refinement. If not visible, error bars are inside the markers.

To evaluate the covalent impact on the system more quantitatively, we first employ a profoundly classical crystal-chemical look at the bonding situation between Pb and its surroundings. The bond-valence sum (BVS) (Brese & O’Keeffe, 1991 ▸) is an easily accessible and empirical measure for the bond capacity of a certain atom within a crystal. It is calculated as the sum over all bond valences of the *j* coordinating atoms with interatomic distances of *r_ij_* as given by
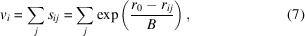
with the standard distance *r*_0_ corresponding to a single bond (2.22 Å for Pb—N) and a scaling length of *B* = 0.37 Å. Fig. 8 ▸ clearly indicates that the BVS of the lead atom increases with increasing pressure (the last pressure point being an exception) due to the shortened Pb—N bond lengths. It is puzzling, however, that the BVS values are generally too large, starting at almost 3; as this is significantly larger than the expected valence for a divalent Pb^II^ atom, the literature parameter needs improvement. Alternatively, one may quantum-chemically determine the BVS from first principles by means of ICOBI as implemented in the *LOBSTER* package. COBI, the crystal orbital bond index, is the periodic equivalent of the Wiberg–Mayer bond index for quantifying bond order in (organic) molecules, and the COBI energy integral (dubbed ICOBI) is the bond order of a pairwise contact. If summed over the entire atomic coordination sphere, this then equals the quantum-chemical BVS. The resulting pressure-dependent ICOBI plot is also given in Fig. 8 ▸, and the ICOBI trend parallels that of the previously shown empirical BVS: upon increasing pressure, the atomic valence increases. One difference is found in the absolute numbers, so ICOBI starts at 1.85 for the lowest pressure point and only exceeds the expected Pb^II^ valence at the highest pressure point, the value being 2.06. Most notably, there is a jump between the third and fourth points, ICOBI increasing from 1.89 to 1.96, exactly where the above-mentioned internal structural change takes place.

The reason for the classical BVS values being too large, as written above, may be that the Pb—N combination implicitly relates to an N^3−^ anion, not the complex NCN^2−^ anion which is less charged, resulting in more covalency and hence a too small ‘single’ bond between Pb and NCN^2−^ and a too large BVS.

**Figure 9:**
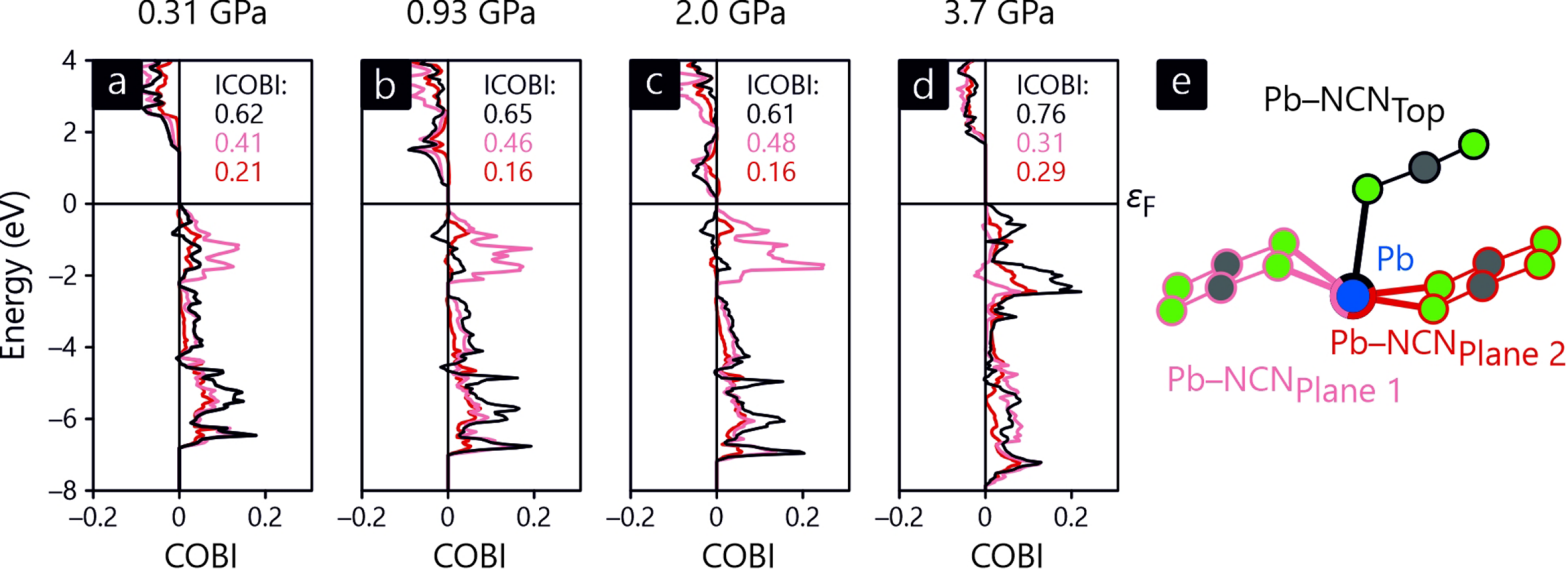
Energy-resolved COBI of the Pb—NCN bonds in PbNCN for (*a*) 0.31 (2) GPa, (*b*) 0.93 (7) GPa, (*c*) 2.0 (2) GPa and (*d*) 3.7 (4) GPa. (*e*) The nearest-neighbor coordination of Pb.

The internal change in the cyanamide unit, seen in both the 1D and 2D refinements, is further analyzed by means of (I)COBI. To do so, the energy-resolved COBI of the Pb—NCN bonds for the first four pressure points are shown in Fig. 9 ▸. Here, panels (*a*) to (*c*) reveal that the shortest and strongest bond, shown in black, suffers from antibonding levels directly below the Fermi level, and this also applies for the longest and weakest bond (light red) but to a lesser extent. At the same time, the band gap decreases during pressurization so that mixing with the strictly antibonding conduction bands leads to further destabilization. Admittedly, the band gaps are notoriously underestimated by DFT, but this does not change the qualitative interpretation. At 3.7 (4) GPa, however, the change in the NCN^2−^ anion’s shape also changes the bonding, leading to stronger bonding below ɛ_F_, as well as an increased band gap separating the bonding valence band from the antibonding conduction band. One may *hypothetically* extrapolate the deformation of the NCN^2−^ anion beyond the 2.0 (2) GPa point, but this leads to a more strongly bent NCN^2−^ anion, thereby both increasing internal anionic stress and weakening the Pb—NCN bond. Hence, nature avoids both by reshuffling the bond multiplicities.

## 4. Conclusions

In this work we have demonstrated that the novel multidimensional Rietveld refinement method used on POWTEX is flexible enough to be extended to other neutron powder diffraction instruments. To do so, an accurate instrument parameter file is required, as small changes may have a big impact on the results, so the *PowderReduceP2d* algorithm, part of the open-source project *Mantid*, was adapted to the slightly different data-reduction workflow required for the SNAP beamline. The changes are publicly available starting with *Mantid* Version 6.8 in order to generate .p2d files for a multidimensional Rietveld refinement.

The first results of a multiphase multidimensional refinement prove not only the technical feasibility but also the method’s beneficial extension to the SNAP instrument. This is especially remarkable considering the small solid-angle coverage – compared with, say, POWTEX – which would not suggest obtaining large differences in the structural details. First, the refined bulk modulus of PbNCN is quite similar to that of related α-PbO. Second, the single-phase multidimensional data indicate an internal change in PbNCN at pressures beyond 2.0 (2) GPa, as also corroborated by electronic structure theory. A quantum-chemical bonding analysis focusing on structural changes between 2.0 (2) and 3.7 (4) GPa highlights subtle but important variations in the bond multiplicities of the NCN^2−^ complex anion. Future investigations of PbNCN single-crystal data may improve the underlying structural models, in particular relating to smaller pressure increments between 2.0 (2) and 3.7 (4) GPa.

## Supplementary Material

28 zipped CIF files for the high-pressure neutron Rietveld refinements, both one- and two-dimensional, for different pressures. DOI: 10.1107/S1600576724007635/tu5048sup1.zip

Supplementary tables and figures. DOI: 10.1107/S1600576724007635/tu5048sup2.pdf

## Figures and Tables

**Table:** The changes for Δ2θ_div_, Δ2θ_detector_, Δ*t*/*t*, Δ*L*/*L* and Δ2θ_sample_ were done based on the instrument characteristics, while DthF is a refined variable.

	*L*_1_ (m)	*L*_2*A*_ (m)	*L*_2*B*_ (rad^−1^)	Δ2θ_div_ (rad)	Δ_add_
Preliminary	15.0	0.5	0.0	0.01769	0.0
Final	15.0	0.5	0.0	0.01769	0.0

**Table:** 

	Δ*t*/*t*	Δ*L*/*L*	DthF	*w*_detector_ (m)	*w*_sample_ (m)
Preliminary	0.0028	0.00026	0.67 (1)	0.001172	0.0076794
Final	0.0025	0.00023	0.854 (4)	0.0004081	0.0036060
