# Supplementary material for: Multidimensional Rietveld refinement of high-pressure neutron diffraction data of PbNCN
Source: J Appl Crystallogr. 2024 Sep 5;57(Pt 5):1436–45. doi: 10.1107/S1600576724007635 (PMC11460395; doi:10.1107/S1600576724007635)
Supplement: Supplementary file 2 [file j-57-01436-sup2.pdf]

JOURNAL OF
APPLIED
CRYSTALLOGRAPHY

**Volume 57 (2024)**

**Supporting information for article:**

# Multidimensional Rietveld refinement of high-pressure neutron diffraction data of PbNCN

**Yannick Meinerzhagen, Katharina Eickmeier, Peter C. Müller, Jan Hempelmann, Andreas Houben and Richard Dronskowski**

**Table S1** Refinement results for the two-phase sample, including the cell volumes of lead and PbNCN for the 1D and 2D refinements as well as the oil pressures and the calculated sample pressures.

| Oil Pressure (bar) | $^{1D}V$(Pb) (Å$^3$) | $^{2D}V$(Pb) (Å$^3$) | $^{1D}$Sample Pressure (GPa) | $^{2D}$Sample Pressure (GPa) | $^{1D}V$(PbNCN) (Å$^3$) | $^{2D}V$(PbNCN) (Å$^3$) |
|---|---|---|---|---|---|---|
| **70** | 123.06(24) | 121.25(11) | 0 | 0.013(3) | 255.70(10) | 252.59(4) |
| **400** | 116.70(19) | 114.84(10) | 2.54(2) | 2.62(2) | 237.93(8) | 234.99(4) |
| **600** | 112.44(18) | 110.50(12) | 4.79(4) | 4.99(3) | 229.06(8) | 226.08(5) |
| **800** | 108.15(25) | 106.38(13) | 7.64(7) | 7.81(7) | 221.9(10) | 219.37(5) |
| **1000** | 103.3(10) | 102.8(4) | 11.73(10) | 10.75(10) | 215.6(4) | 212.81(16) |

**Table S2** As before, but for the single-phase sample of PbNCN.

| Oil Pressure (bar) | $^{1D}V$(PbNCN) (Å$^3$) | $^{1D}$Sample Pressure (GPa) | $^{2D}V$(PbNCN) (Å$^3$) | $^{2D}$Sample Pressure (GPa) |
|---|---|---|---|---|
| **70** | 252.65(20) | 0.25(2) | 249.72(3) | 0.31(2) |
| **150** | 247.66(19) | 0.77(7) | 244.83(3) | 0.93(7) |
| **250** | 240.93(20) | 1.7(2) | 238.36(3) | 2.0(2) |
| **400** | 233.49(18) | 3.4(4) | 230.68(3) | 3.7(4) |
| **500** | 229.62(18) | 4.6(6) | 226.81(3) | 4.9(5) |
| **600** | 226.12(20) | 5.9(7) | 223.68(4) | 5.9(7) |
| **700** | 222.87(23) | 7.3(10) | 220.07(4) | 7.3(9) |
| **800** | 220.35(23) | 8.6(12) | 217.53(4) | 8.4(10) |
| **900** | 218.00(29) | 10.0(14) | 215.21(5) | 9.5(12) |

**Table S3** Refined spatial parameters, all atoms on Wyckoff position 4*c*, $y \equiv ¼$. The top row always shows the 1D values, while the bottom row always shows the 2D values. Atomic displacement parameters were not refined, i.e., $U_{iso} = 0$.

| Oil Pressure | Pb | | N1 | | N2 | | C | |
|---|---|---|---|---|---|---|---|---|
| (bar) | *x* | *z* | *x* | *z* | *x* | *z* | *x* | *z* |
| **70** | 0.3905(22) | 0.6368(8) | 0.3465(22) | 0.4587(9) | 0.9018(21) | 0.3640(10) | 0.1593(26) | 0.4042(10) |
| | 0.3942(11) | 0.6311(5) | 0.3298(12) | 0.4573(5) | 0.9041(12) | 0.3605(5) | 0.1438(15) | 0.4022(7) |
| **150** | 0.3876(20) | 0.6398(7) | 0.3439(21) | 0.4673(8) | 0.9002(19) | 0.3665(9) | 0.1764(23) | 0.4022(11) |
| | 0.3913(10) | 0.6394(5) | 0.3192(11) | 0.4692(5) | 0.8962(11) | 0.3699(5) | 0.1622(12) | 0.3966(7) |
| **250** | 0.3910(22) | 0.6409(8) | 0.3452(24) | 0.4700(9) | 0.9022(21) | 0.3644(10) | 0.1854(24) | 0.4025(13) |
| | 0.3954(10) | 0.6446(5) | 0.3122(11) | 0.4717(5) | 0.8870(10) | 0.3721(5) | 0.1739(11) | 0.3913(8) |
| **400** | 0.3644(21) | 0.6355(7) | 0.3613(21) | 0.4602(9) | 0.9087(20) | 0.3651(9) | 0.109(28) | 0.4258(9) |
| | 0.3729(11) | 0.6332(4) | 0.3621(13) | 0.4522(5) | 0.9163(10) | 0.3637(5) | 0.0996(15) | 0.4183(6) |
| **500** | 0.3694(20) | 0.6382(7) | 0.3683(21) | 0.4627(10) | 0.9132(19) | 0.3681(9) | 0.1061(28) | 0.4272(10) |
| | 0.3705(10) | 0.6347(5) | 0.3747(12) | 0.4521(6) | 0.9216(10) | 0.3657(6) | 0.0968(15) | 0.4202(6) |
| **600** | 0.3691(20) | 0.6383(8) | 0.3753(22) | 0.4642(10) | 0.9142(19) | 0.3687(10) | 0.0986(28) | 0.4264(12) |
| | 0.3659(11) | 0.6372(6) | 0.3843(13) | 0.4558(8) | 0.9211(11) | 0.3671(7) | 0.0927(17) | 0.4215(7) |
| **700** | 0.3681(20) | 0.6354(9) | 0.3820(22) | 0.4624(12) | 0.9127(20) | 0.3661(11) | 0.0909(29) | 0.4239(13) |
| | 0.3720(13) | 0.6354(6) | 0.3866(14) | 0.4541(9) | 0.9190(12) | 0.3705(7) | 0.0909(18) | 0.4274(8) |
| **800** | 0.3662(21) | 0.6210(11) | 0.3878(21) | 0.4466(14) | 0.9066(23) | 0.3542(11) | 0.0749(27) | 0.4208(15) |
| | 0.3749(13) | 0.6312(7) | 0.3823(14) | 0.4469(8) | 0.9134(14) | 0.3635(8) | 0.0751(18) | 0.4246(10) |
| **900** | 0.3723(26) | 0.6283(13) | 0.3933(27) | 0.4434(16) | 0.9007(29) | 0.3388(12) | 0.061(3) | 0.4119(21) |
| | 0.3824(15) | 0.6326(8) | 0.3810(17) | 0.4382(10) | 0.9094(17) | 0.3484(8) | 0.058(2) | 0.4195(14) |

**Table S4** Refined cell parameters of PbNCN from single-phase refinements. The top row refers to the 1D values, while the bottom row refers to the 2D values.

| $^{2D}$Sample Pressure (GPa) | Oil Pressure (bar) | *a* (Å) | *b* (Å) | *c* (Å) |
|---|---|---|---|---|
| **0.31(2)** | 70 | 5.5732(19) | 3.8710(9) | 11.7111(24) |
| | | 5.5527(3) | 3.8553(2) | 11.6651(3) |
| **0.93(7)** | 150 | 5.5654(18) | 3.8471(9) | 11.5673(26) |
| | | 5.5470(3) | 3.8322(2) | 11.5179(4) |
| **2.0(2)** | 250 | 5.5531(18) | 3.8197(9) | 11.3588(31) |
| | | 5.5377(3) | 3.8037(2) | 11.3167(4) |
| **3.8(4)** | 400 | 5.5576(18) | 3.7874(8) | 11.0931(27) |
| | | 5.5359(3) | 3.7724(1) | 11.0465(2) |
| **4.9(5)** | 500 | 5.5554(18) | 3.7695(8) | 10.9651(28) |

| | | | | |
|---|---|---|---|---|
| | | 5.5336(3) | 3.7512(2) | 10.9271(4) |
| **5.9(7)** | 600 | 5.5494(20) | 3.7576(9) | 10.8438(32) |
| | | 5.5279(3) | 3.7337(2) | 10.8377(5) |
| **7.3(9)** | 700 | 5.5417(21) | 3.7508(11) | 10.722(4) |
| | | 5.5223(4) | 3.7430(2) | 10.6471(5) |
| **8.4(10)** | 800 | 5.5361(21) | 3.7455(11) | 10.626(4) |
| | | 5.5131(4) | 3.7355(2) | 10.5629(6) |
| **9.5(12)** | 900 | 5.5241(26) | 3.7411(14) | 10.549(5) |
| | | 5.5041(4) | 3.7259(2) | 10.4939(6) |

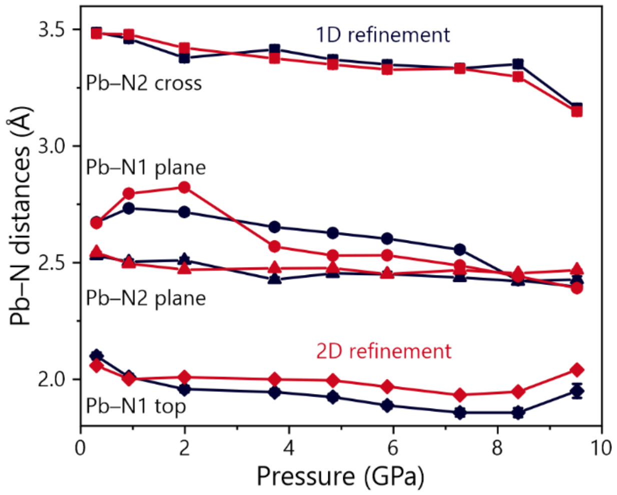


**Figure S1** Development of the Pb–N distances with increasing pressure.

During the initial data reduction for 2D data sets we noticed a peculiar intensity variation with the $2\theta$ angle: the intensities of all data points below 63° were collectively lower. The reason was found in the initial mask used for the data reduction. As shown in the top part of Figure S2, one of the detectors only collected data in two of the three in-plane detector modules, while the other detector collected data in all three in-plane modules, so the abrupt change of intensity at $2\theta \approx 63°$ is easy to comprehend. The problem was solved by masking one detector completely and using only data collected by the other detector. The new mask can be seen in the lower part of Figure S2 and was applied to both the 2D and the conventional data reduction to ensure comparability. Except for this, the patterns for the conventional Rietveld refinement were reduced following the standard SNAP data reduction procedure.

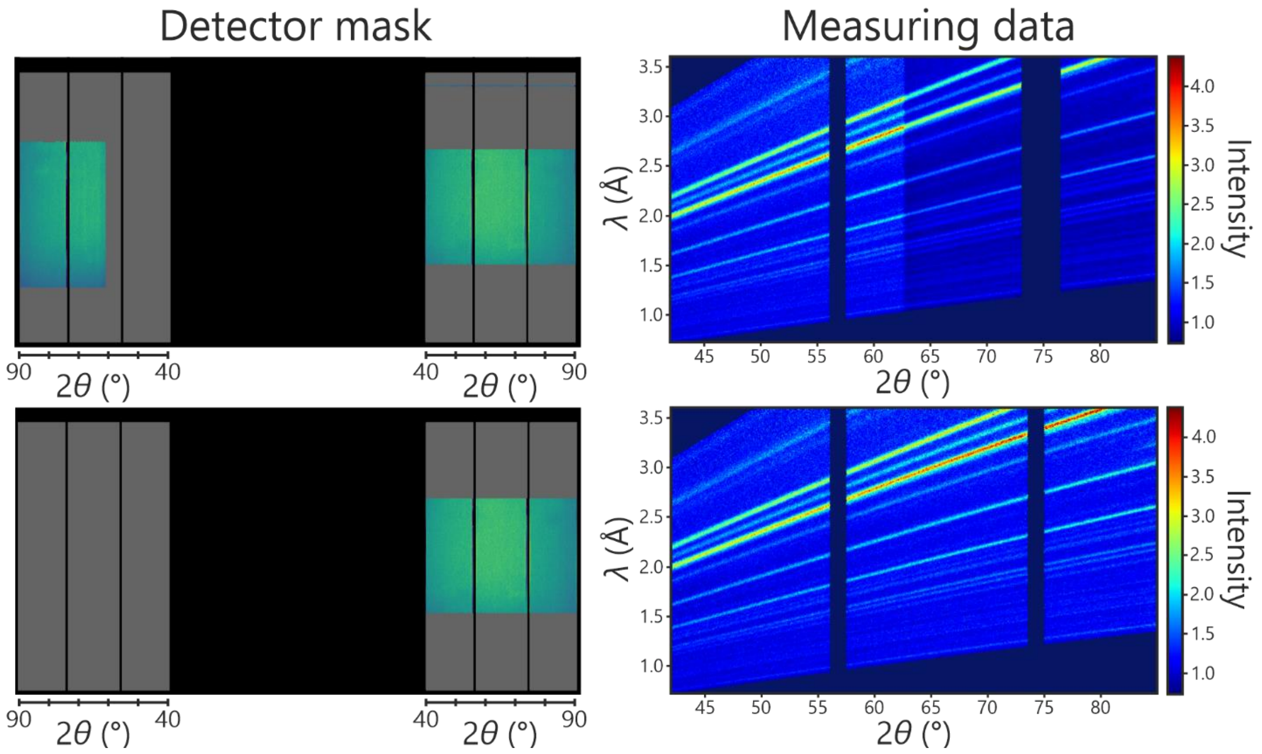


**Figure S2** Top left is a view of the detector from the Mantid software after masking. Top right is the associated multidimensional data set with the conspicuous intensity change at around 63°. Bottom left is the detector view with the adapted mask to only use one of the detectors. Bottom right is the associated multidimensional data set without an intensity variation.
